# Expression of Apelin in Rotator Cuff Tears and Examination of Its Regulatory Mechanism: A Translational Study

**DOI:** 10.7759/cureus.44347

**Published:** 2023-08-29

**Authors:** Mitsufumi Nakawaki, Tomonori Kenmoku, Kentaro Uchida, Lars Arendt-Nielsen, Naoshige Nagura, Masashi Takaso

**Affiliations:** 1 Orthopaedic Surgery, Kitasato University School of Medicine, Sagamihara, JPN; 2 Orthopaedic Surgery, Kitasato University Hospital, Sagamihara, JPN; 3 Health Science and Technology, Center for Neuroplasticity and Pain (CNP), Faculty of Medicine, Aalborg University, Aalborg, DNK; 4 Health Science and Technology, Center for Sensory-Motor Interaction (SMI), Faculty of Medicine, Aalborg University, Aalborg, DNK

**Keywords:** rotator cuff tear, pain, tumor necrosis factor α, inflammatory mediators, apelin

## Abstract

Objectives: Inflammatory mediators play important roles in the pain associated with rotator cuff tears (RCTs), but their underlying mechanisms are unclear. Apelin, a neuropeptide, is upregulated under inflammatory conditions and possibly contributes to pain induced by rotator cuff tears. This translational study aimed to examine apelin expression and regulation by tumor necrosis factor alpha (TNF-α) in patients with RCT and in rat RCT models.

Methods: Synovial tissues were harvested from the glenohumeral joints of the shoulders in 46 patients who underwent arthroscopic Bankart repair for recurrent shoulder dislocations (RSDs) or arthroscopic rotator cuff repair for RCTs. The harvested tissues were extracted and processed by reverse transcriptase-polymerase chain reaction (RT-PCR). Rats underwent sham or RCT surgery; the rotator cuff tissues were extracted 1, 7, 14, 28, and 56 days after surgery and analyzed for mRNA expression levels of the TNF-α and apelin using RT-PCR. The cultured rotator cuff cells (RCCs) were stimulated with TNF-α to examine their role in the regulation of apelin expression.

Results: Apelin expression was higher in the RCT group than in the RSD group and significantly correlated with pain intensity. In rats, the expression was also higher in RCT. Apelin expression significantly increased during the acute and chronic phases in rats.

Conclusions: In cultured RCCs, apelin mRNA levels significantly increased after TNF-α stimulation. Apelin levels were regulated by TNF-α and were highly expressed in patients with RCT and rats in RCT models. Thus, apelin may be a new pain management target for RCTs.

## Introduction

Rotator cuff tears (RCTs) are frequently associated with shoulder dysfunction and pain. They are common in older patients, with an incidence of 46% in those aged 70 years and above [1]. In patients with RCTs, the pain is generally triggered by movements, especially at night, causing a decline in activities of daily living and poor quality of life. However, the prevalence of symptomatic RCTs is approximately half that of asymptomatic RCTs [2]. While the involvement and regulatory mechanisms of inflammatory mediators in pain associated with RCTs have been investigated, the mechanism governing the development of pain in patients with RCTs remains unknown.

Recent reports have shown that neuropeptides are upregulated in musculoskeletal tissues, such as bones, muscles, periosteum, and synovial tissues, under inflammatory conditions and may contribute to pain [3-5]. Apelin, a neuropeptide, is the specific endogenous ligand of the G protein-coupled receptor, APJ. It is synthesized as a preproprotein comprising 77 amino acid residues. The final functionally active protein is formed by processing the C-terminal fragment of the preprotein. Apelin and its receptor (APJ) are expressed in the central and peripheral nervous systems and are linked to various neuropathic pain processes [6-9]. However, the dynamics of apelin expression in RCT have not been elucidated. Tumor necrosis factor alpha (TNF-α) is a major inflammatory cytokine in RCTs and is being overexpressed in patients with RCTs compared to healthy individuals (controls) [10]. In a rat RCT model, RCT repair resulted in reduced TNF-α expression and pain attenuation [11]. 

In a previous study, we reported that TNF-α levels remained constantly elevated in a rat RCT model for 56 days [12]. Another study reported the presence of TNF-α-stimulated apelin in adipocytes of humans and mice [13]. However, the regulation of apelin by TNF-α in patients with RCT or rat RCT models has not been investigated. The present translational study aimed to examine apelin expression and regulation by TNF-α in patients with RCT and a rat RCT model.

## Materials and methods

Participants and specimens

The study was conducted per the guidelines of the Ethics Review Committee of Kitasato University (approval no. KMEO B13-113). Patients included in the study provided written informed consent for participation. To investigate apelin expression in RCTs, synovial tissues of the glenohumeral joint were harvested from 23 patients during arthroscopic rotator cuff repair (for painful degenerative RCTs). Two types of tissue were collected: one from the most inflamed synovium of the rotator interval (RI group) and the other from the subacromial bursa around the coracoacromial ligament (SAB group), where the greater tuberosity of the humeral head usually impinges. We also obtained synovial tissues around the rotator intervals from the shoulders of 23 patients during arthroscopic Bankart repair for recurrent shoulder dislocations (RSD group). The RSD group was used as the control group because these patients experience less or no pain during daily activities and thus may present different pain mechanisms. The inclusion criterion for patients was the onset of shoulder pain more than one month before surgery. The exclusion criteria were the presence of chronic systemic diseases, such as rheumatism and kidney disease, shoulder fractures at the time of surgery, previous shoulder operations, and corticosteroid usage initiated one month before surgery. The numeric rating scale (NRS) was used to evaluate the severity of shoulder pain in each patient. 

All arthroscopic surgeries were performed by a single surgeon (K.T.) who has 10 years of experience. We harvested the synovial membrane samples from the anterior side of the glenohumeral joints. Synovial tissues were extracted and processed for reverse transcriptase-polymerase chain reaction (RT-PCR). 

Rat RCT models

The study with a rat model was designed and conducted per the guidelines of the Ethics Review Committee of Kitasato University for Animal Experimentation (approval no. 2018-090). We used a total of 85 male Sprague-Dawley rats (nine weeks old; Charles River Laboratories, Inc., Tokyo, Japan) in this study. Eighty rats were randomly divided into control and RCT groups (n = 40). Surgeries to induce RCTs were conducted under general anesthesia with 500 μL/kg of a mixture of Domitor (Nippon Zenyaku Co., Fukushima, Japan), Midazolam (Sand Co., Yamagata, Japan), and Vetorphale (Meiji Seika Co., Tokyo, Japan) at a ratio of 3:1:1. To induce RCT in rats (RCT group), a longitudinal skin incision was made in the right shoulder, and the deltoid muscle was split to reveal the rotator cuff tendons. The supraspinatus and infraspinatus tendons were transected entirely, and the skin was closed using 5-0 nylon sutures (Natsume, Tokyo, Japan). The control group underwent the same surgical procedure on the left shoulder, except for the transection of the infraspinatus and supraspinatus tendons. The rats were allowed to recover and regain activity without restriction after surgery. The surgically treated rotator cuff tissues were extracted from the control and RCT groups (n = 8 from each group) at 1, 7, 14, 28, and 56 days after surgery and processed for RT-PCR. Further, rotator cuff tissues from five normal rats were used for cell culture studies.

Reverse transcriptase-polymerase chain reaction

The RNA extraction, complementary DNA synthesis, and RT-PCR using extracted rotator cuff tissues were performed as described previously [12]. Primer pairs for quantitative PCR are shown in Table 1. 

**Table 1:** Sequences of the primers used in this study GAPDH: Glyceraldehyde-3-phosphate dehydrogenase, TNF-α: Tumor necrosis factor alpha

Primer	Sequence (5'–3')	Product size (bp)
Apelin (human)-sense	GAA TCT GCG GCT CTG CGT	76
Apelin (human)-antisense	CAT CAG GGA CCC TCC ACA CA	
GAPDH (human)-sense	TGT TGC CAT CAA TGA CCC CTT	223
GAPDH (human)-antisense	CTC CAC GAC GTA CTC AGC G	
TNF-α (mouse)-sense	CTC TTC TCA TTC CCG CT CGT	104
TNF-α (mouse)-antisense	GGG AGC CCA TTT GGG AAC TT	
Apelin (mouse)-sense	CTT GAC TGC CGT GTG TGG A	72
Apelin (mouse)-antisense	5ʹ-CGC ATG TTG CCT TCT AGC	
GAPDH (mouse)-sense	TGC CAC TCA GAA GAC TGT GG	129
GAPDH (mouse)-antisense	TTC AGC TCT GGG ATG ACC TT	

The expression levels of the mRNA of the genes of interest were normalized to those of glyceraldehyde-3-phosphate dehydrogenase (GAPDH). In particular, we measured the expression levels of apelin in the human synovial tissues and compared the results of the RSD, RCT-RI, and RCT-SAB groups. In addition, we measured the expression levels of the inflammatory mediator TNF-α and the neuropeptide apelin in the tissue samples and compared the results between the control and RCT groups at different time points.

Rotator cuff cell (RCC) culture

Seven days after surgery, the rotator cuff tissues from nine-week-old Sprague-Dawley rats (n = 5) were digested using 15 mL of 1 mg/mL collagenase to extract RCCs. The RCCs were cultured in α-minimal essential media (Thermo Scientiﬁc, Waltham, MA, USA) supplemented with 10% fetal bovine serum in six-well plates for 7 days. Subsequently, the RCCs were stimulated with vehicle (serum-free α-minimal essential media) or 10 ng/mL rat recombinant TNF-α (BioLegend, San Diego, CA, USA) for 24 hours. Total mRNA was extracted and examined by RT-PCR.

Statistical analysis

Statistical analyses were performed using JMP Pro 16.1 (SAS Institute Inc., Cary, NC, USA). Age and sex were assessed using the Student’s t-test and chi-square test, respectively. Differences in gene expression among the groups RCT-SAB, RCT-RI, and RSD were compared using analysis of variance (ANOVA). A post-hoc analysis of the differences in expression measured at different time points was performed using Dunnett’s test. Correlations between apelin expression in the synovium of the glenohumeral joint and NRS and age were determined using Spearman’s rank correlation coefficient. Changes in the expression of inflammatory cytokines and neuropeptides in the rotator cuff tissues were also analyzed in the rat RCT models using ANOVA. A post-hoc analysis of differences in expression measured at different time points was performed using Tukey’s honestly significant difference test. A p-value of <0.05 was considered to indicate statistical significance. The classification scheme for the correlation was defined as follows: 0 < |ρ| < 0.2, negligible; 0.2 < |ρ| < 0.4, low; 0.4 < |ρ| < 0.7, moderate; 0.7 < |ρ|, high.

## Results

Comparison of RCT and RSD groups 

**Figure 1:**
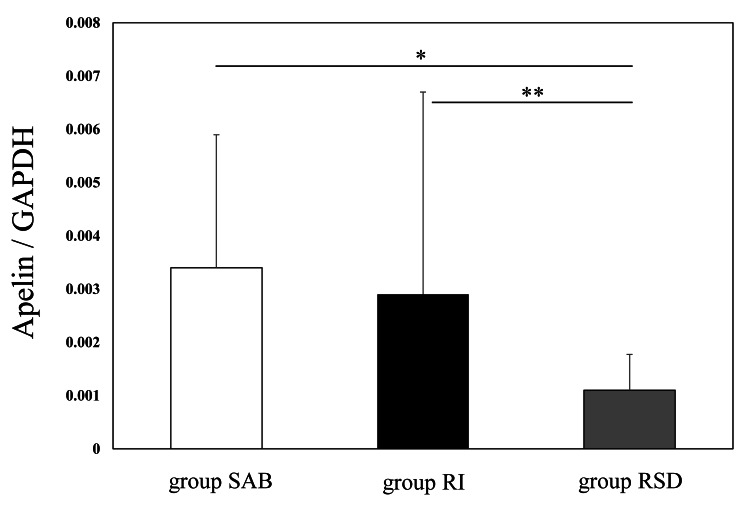
Apelin messenger mRNA expression in RSD and RCT This graph displays the qPCR analysis of apelin mRNA expression in the RCT and RSD groups (n = 23). White box: Group SAB, 0.0034 ± 0.0025; Black box: Group RI, 0.0029 ± 0.0037; Gray box: Group RSD, 0.0011 ± 0.0007 ANOVA, p = 0.01; post-hoc test *p = 0.019, **p = 0.014 RCTs: Rotator cuff tears; RSD: Recurrent shoulder dislocation; SAB: Subacromial bursa; RI: Rotator interval; qPCR: Quantitative polymerase chain reaction; GAPDH: Glyceraldehyde-3-phosphate dehydrogenase

The RCT group consisted of 23 samples (16 from the dominant side of the shoulder and 7 from the non-dominant side). The mean constant score was 39.4 ± 17.7 points (range, 12-69) [14]. The RSD group likewise comprised 23 samples (13 from the dominant side and 10 from the non-dominant side). The mean Rowe score was 49.6 ± 12.2 points (range, 25-60) [15]. Age was significantly higher in the RCT group than in the RSD group (65.0 ± 10.6 vs. 26.3 ± 11.7 years; p <0.001), but there was no significant difference in the sex ratio (15 men, 8 women vs. 18 men, 5 women; p = 0.51). Apelin* *expression was significantly higher in the RCT group than in the RSD group (p = 0.01). The post-hoc test revealed that both the RCT groups, SAB and RI, showed significantly higher apelin expression than the RSD group (p = 0.009 and 0.04, respectively; Figure 1). 

Correlation between apelin expression and pain

**Figure 2:**
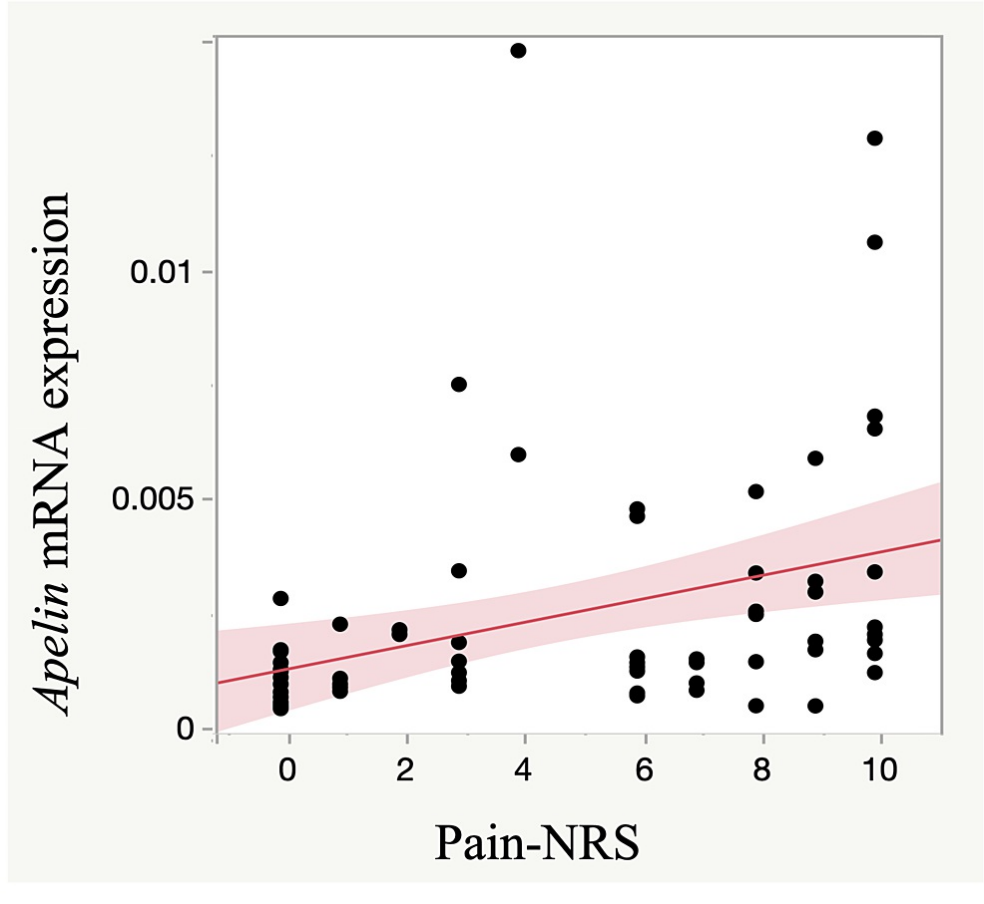
Correlation between apelin expression and NRS There was a significant correlation between apelin expression and NRS-pain (ρ = 0.49, p <0.001). The red line indicates the regression line. NRS: Numeric rating scale

The mean pain intensity NRS of the RCT and RSD groups were 6.3 ± 3.1 and 0.2 ± 0.4, respectively. In addition, there was a moderate but significant correlation between apelin expression and NRS (ρ = 0.49, p <0.001; Figure 2).

Correlation between apelin expression and age

**Figure 3:**
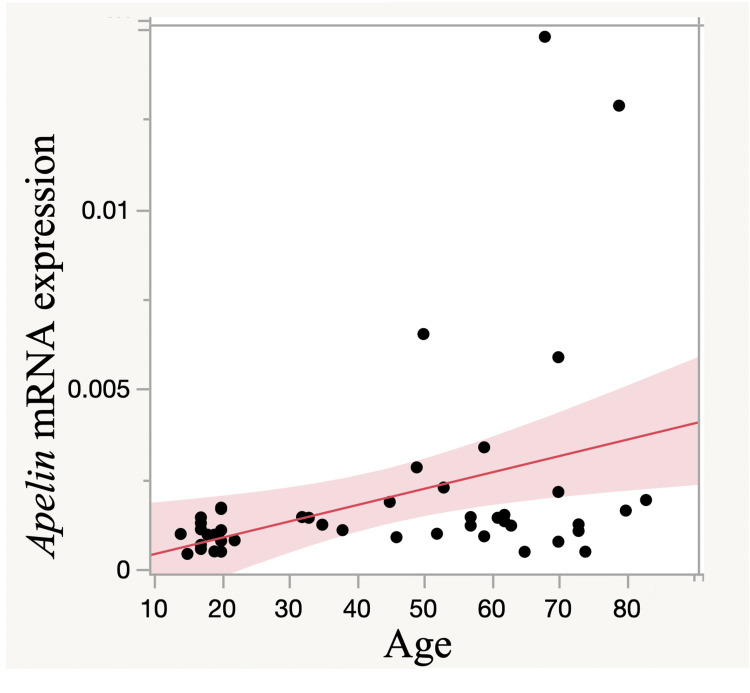
Correlation between apelin expression and age There was a significant moderate correlation between apelin expression in RI and NRS (ρ = 0.39, p = 0.005). The red line indicates the regression line. RI: Rotator interval; NRS: Numeric rating scale

There was a moderate but significant correlation between apelin expression in RI and NRS (ρ = 0.39, p = 0.005; Figure 3).

Gross appearance of rotator cuff tissues after RCT repair in rats 

No continuity of the rotator cuff was observed on postoperative day 1. Fibrous tissue was observed covering the transected area 7 days after surgery. After 14 days, the humeral head of the rat was covered by fibrous tissue.

TNF-α and apelin mRNA expression after RCT repair in rats

**Figure 4:**
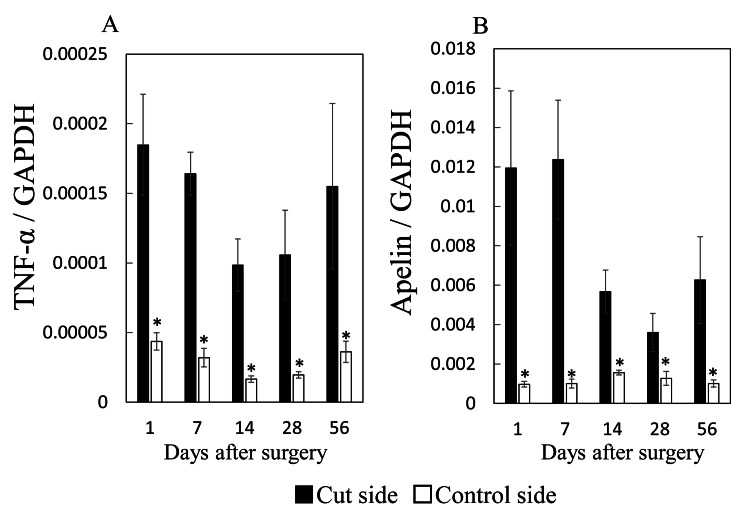
The qPCR analysis of TNF-α (A) and apelin (B) mRNA expression in a rat RCT model Data are represented as mean ± standard error (n = 8 from each group). *p <0.05 versus control group. qPCR: Quantitative polymerase chain reaction; TNF-α: Tumor necrosis factor alpha; RCT: Rotator cuff tear; GAPDH: Glyceraldehyde-3-phosphate dehydrogenase

The TNF-α mRNA levels were significantly higher in the RCT groups than in the control group at all time points (1 day, p = 0.002; 7 days, p <0.001; 14 days, p <0.001; 28 days, p = 0.019; 56 days, p = 0.049). In contrast, TNF-α continued to be expressed without significant differences in expression levels between measurement time points (p = 0.375) (Figure 4A). Apelin expression was significantly higher in the RCT group than in the control group at all time points after surgery (p = 0.004, 0.001, 0.001, 0.046, and 0.004 at 1, 7, 14, 28, and 56 days, respectively) (Figure 4B). The ANOVA demonstrated significant changes in expression across the measured time points for apelin (p = 0.028). Post-hoc analysis, however, showed no significant change in apelin mRNA levels with time. 

Effects of TNF-α on apelin expression in RCCs in rats

**Figure 5:**
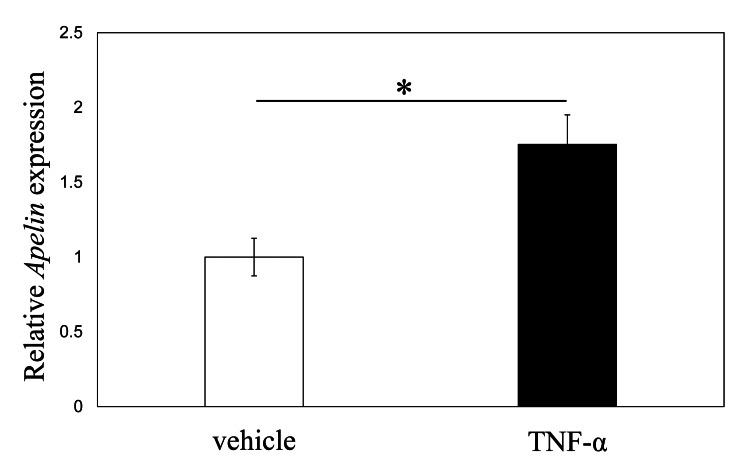
Effect of TNF-α on apelin mRNA expression in RCCs The qPCR analysis of apelin mRNA expression in RCCs. Data are represented as mean ± standard error (n = 8). *p <0.05 versus vehicle group. qPCR: Quantitative polymerase chain reaction; TNF-α: Tumor necrosis factor alpha; RCC: Rotator cuff cell

The RT-PCR analysis showed that stimulation of RCCs with rat recombinant TNF-α resulted in a 1.8-fold significant increase in apelin mRNA expression (p = 0.003; Figure 5). 

## Discussion

This translational study showed that in contrast to the non-painful recurrent shoulder dislocation group, the groups with painful RCTs had considerably increased apelin expression. Moreover, in rats, the rotator cuff tissues in the painful RCT groups showed increased levels of apelin mRNA expression compared with the control group. The TNF-α expression patterns are consistent with those in our previous report [12]. Stimulation of cultured RCCs with TNF-α altered apelin expression. These findings provide insight into the possible pain mechanism mediated by the inflammatory mediator TNF-α and provide new useful insight for the identification of new therapeutic targets for pain management in patients with painful RCTs.

Apelin contributes to several physiological and cellular events, such as angiogenesis [16], maintenance of tissue homeostasis [17], and tissue remodeling in aging or tissue injury [18]. The healing potential of the rotator cuff is considered poor because the degenerative RCT area is vulnerable to activities of daily living and the tendon origin is a hypovascular area [19]. Therefore, apelin expression may be enhanced for RCT repair, much like other inflammatory cytokines and growth factors linked to pain and tissue remodeling [20-23]. So, more research is required to identify the limits or threshold values that determine whether these molecules are likely to participate in the process of pain or contribute to tissue repair.

Possible mechanisms 

The SAB is susceptible to mechanical stress in shoulders with RCTs because of subacromial impingement during activities involving the upper extremities. In addition, pain induced by RCTs is associated with subacromial bursitis more than synovitis in the glenohumeral joint [24,25]. Therefore, apelin expression in SAB was significantly higher than that of RI in patients with RCTs. 

The production of apelin, induced by muscle contraction, decreases in an age-dependent manner; thus, apelin is positively associated with the beneficial effects of exercise and can reverse age-associated sarcopenia in older persons [17]. Thus, we had supposed that apelin expression in the RCT group, with a higher mean age, would be significantly lower than that in the RSD group. However, apelin expression was significantly higher in the synovium derived from patients with painful RCTs than in patients with non-painful RSD. In addition, this study showed a moderate correlation between apelin expression and age. Therefore, our results are not consistent with those of the previous report. Apelin is expressed in several pathophysiological conditions such as osteoarthritis [5], obesity, diabetes mellitus, and diabetes-related diseases [26,27]. In addition, this study found a moderate correlation between apelin expression and pain. Apelin expression continued to increase across the measured time points after the rotator cuff injury in rats, suggesting that rotator cuff injuries are associated with increased apelin expression. Apelin expression in the SAB, which is vulnerable to subacromial impingement, is significantly increased compared to RI patients. Thus, RCTs can explain our controversial data on age. 

Interaction between TNF-α and apelin

Several studies have suggested that increased TNF-α expression is related to pain in RCTs [10,11,17]. The TNF-α expression in patients with symptomatic RCTs and pain was higher than in patients with anterior shoulder instabilities who did not have severe shoulder pain [17]. In a rat RCT model, the repair of RCT reduced TNF-α expression and attenuated nociception [11]. In the present study with a rat RCT model, apelin was continuously expressed after RCT, consistent with the TNF-α expression pattern. In addition, apelin expression significantly increased in RCCs after TNF-α treatment. These results imply that apelin may be induced by TNF-α and contribute to TNF-α-mediated RCT.

Apelin and its receptor system (APJ) are located in the central and peripheral nervous systems [6,8]. Earlier studies have shown that intrathecal injection of apelin induces hyperalgesia [7], and peripheral injection of apelin-13 enhances pain sensitivity [28]. In addition, an APJ antagonist was reported to reduce chronic constriction injury-induced hyperalgesia [9]. According to the present and previous results, apelin expression induced by TNF-α in the synovium of painful RCTs is thought to be associated with RCT pain, especially pain sensitivity. Our results imply that apelin expression can be a therapeutic target for pain control in RCT. Therefore, further investigations are warranted to elucidate the pain mechanism in RCT.

Limitations

This study has some limitations. First, we could not show a direct causative correlation between pain and apelin levels. Further investigation is needed to determine whether elevated apelin levels contribute to pain in RCTs. Second, we did not determine the protein levels in rotator cuff tissues. Finally, our study using human synovial tissues lacked control data from healthy individuals. Third, this study was a cross-sectional study, so we could not assess the change in apelin after healing. However, it is not possible to perform a second surgery only to obtain synovial tissue due to ethical issues. Thus, in this study, we could not determine the changes in apelin levels over time in humans. Nevertheless, the results of our study do indicate an association between apelin expression and TNF-α in RCT and that apelin expression can be a useful therapeutic target for pain control in RCT.

## Conclusions

Apelin expression was higher in the painful RCT group than in the RSD group in humans, especially in the SAB, and was associated with pain intensity. Apelin levels were also elevated in the rat RCT model and were significantly and continuously elevated for 56 days after RCT injury, indicating that apelin expression was possibly induced by TNF-α. Further investigation may reveal the role of apelin in RCTs under an inflammatory state and that targeting apelin may be a new option for managing pain in RCTs.
